# The Efficacy of Bipolar Sealer on Blood Loss in Primary Total Hip Arthroplasty: A Meta-Analysis

**DOI:** 10.1097/MD.0000000000003435

**Published:** 2016-05-13

**Authors:** Ji-Kang Min, Qiang-Hua Zhang, Hai-Dong Li, Heng Li, Pan Guo

**Affiliations:** From the Department of Orthopaedics, The First People's Hospital of Huzhou, Huzhou, Zhe Jiang Province, China.

## Abstract

The blood loss during total hip arthroplasty is difficult to manage and there is no consensus about the effect of bipolar sealer used during operation. Thus, a systematic review of randomized controlled trials (RCTs) was performed to evaluate the efficacy and safety of blood loss using bipolar sealer after total hip arthroplasty (THA).

Relevant literature of comparisons of bipolar sealer after THA for blood loss were searched for in Embase, PubMed, Web of Science, the Cochrane Central Register of Controlled Trials, and Google scholar from their inception to October, 2015. High-quality RCTs were selected to evaluate the need for transfusion, blood loss, and other complications. The software RevMan 5.30 was used for the meta-analysis.

Six studies reporting on 6 RCTs comprising 751 patients were included. Compared with standard electrocautery, bipolar sealer was associated with lower rates of need for transfusion (relative risk [RR] = 0.60; 95% confidence interval [CI] 0.39–0.94), estimated blood loss (mean differences [MD] = −127.39; 95% CI −233.32 to −21.46; *P* = 0.02), and lower total blood loss (MD = −226.57; 95% CI −350.80–102.34; *P* = 0.0004). There is no significant difference between the hemoglobin drop, blood loss in drainage, intraoperative blood loss, Harris score, and rates of infection.

The present meta-analysis indicated that bipolar sealer can decrease the need for transfusion and total blood loss; however, there is no benefit of bipolar sealer from the recovery. It is still need for samples to determine the balance between the economic cost and transfusion.

## INTRODUCTION

Total hip arthroplasty (THA) is associated with a large amount of blood loss and a high transfusion rate.^1^ It has been demonstrated that the blood loss during THA ranging from 700 to 2000 mL and the transfusion rate is 16% to 37%.^2–4^ The blood loss and subsequently transfusion will increase the mortality, length of hospital stay, and add additional cost to patients. Therefore, effective control of blood loss after THA is of extreme importance for the postoperative management of these patients and improve patient satisfaction.

Several hemostasis strategies have been used to reduce blood loss in THA including topical and intravenous administration tranexamic acid, fibrin sealant, bipolar sealer, or electrocautery.^5–8^ However, compared with electrocautery, a new blood loss device, bipolar sealer, which provided hemostasis at lower temperature (<100 °C) and thus, can decrease severe burns, severe tissue necrosis, and operating room fires. Since the bipolar sealer delivers radiofrequency energy to saline for hemostatic sealing and coagulation of soft tissue, and is currently utilized in total knee replacement, total hip arthroplasty, and hepatic transplantations. However, different studies draw contradict conclusion with each other, and a meta-analysis draw a conclusion that the bipolar sealer cannot reduce the need for transfusion and intraoperative blood loss.

With more randomized controlled trials (RCT) have been published, it is necessary to reassess the effect of bipolar sealer in reducing blood loss during THA. Thus, we carried out a meta-analysis to compare the existing evidence and determine whether there were any differences between bipolar sealer and electrocautery in terms of need for transfusion, total blood loss, blood loss in drainage, hidden blood loss, and rate of infection.

## MATERIALS AND METHODS

Ethical approval for this study was not unnecessary since it was a meta-analysis that collect and analysis data from the existing literatures.

### Search Strategy

Electronic databases, including Medline, Embase, PubMed, Cochrane Controlled Trials Register, Web of Science, and Google Scholar, were searched in September, 2015 to identify relevant studies comparing the use of bipolar sealer and electrocautery in the management of blood loss after THA. The keywords and medical subject heading (Mesh) terms used for the search were “bipolar sealer,” “electrocautery,” “total hip arthroplasty,” “total hip replacement,” “THA,” and “THR” were combined with Boolean operators AND or OR. Additionally, the reference lists of all the full-text articles were reviewed to identify any initially omitted studies. There were no restrictions regarding the language of the publications.

### Eligibility Criteria and Study Quality

Study selection was performed according to the following inclusive criteria: studies of patients undergoing primary THA intervention; with bipolar sealer and standard electrocautery as hemostatic strategies to reduce blood loss during THA; studies assessing primary outcomes such as need for transfusion, total blood loss, blood loss in drainage, intraoperative blood loss, the second blood loss such as Harris score, the occurrence rate of infection; and studies designed as RCTs. All the studies were required to be clinical trials. Trials on cadavers or artificial models were excluded. Letters, comments, editorials, and practice guidelines were also excluded.

The Cochrane Handbook for Systematic Reviews of Interventions was used to evaluate the methodological quality and risk bias of the included studies, according to the following parameters: method of randomization, allocation concealment, appropriateness of blinding, and completeness of outcome data. Discrepancies were resolved by consensus after discussion, and a 3rd reviewer participated in the debates to determine the final outcomes, if necessary.

### Data Extraction

Once the duplicates were excluded, 2 reviewers (J-KM and Q-HZ) independently read the titles and abstracts of the literature. Most of the articles can be removed based on the topic of the article and disagreements were solved by discussion or go back to the original article to identify whether or not include it. Data in other forms (i.e., median, interquartile range, and mean ± 95% confidence interval [CI]) were converted to mean ± SD according to Cochrane Handbook. If the data were not reported numerically, we extracted them by Software “Getdata Graph Digitizer” from the published figures.

The following data were extracted and written in the excel: patient demographic data such as author's name, publication date, sample size of the bipolar sealer and control group, location of study, ratio of male and female subjects, operative approach, and prosthesis type of hip; transfusion criteria; and prophylactic antithrombotic and the length of follow-up.

### Outcome Measures and Statistical Analysis

The main outcomes were need for transfusion, total blood loss, blood loss in drainage, hidden blood loss, and intraoperative blood loss. The 2nd outcome measures were length of hospital stay and the rates of infection. Continuous outcomes (hemoglobin drop, total blood loss, blood loss in drainage, hidden blood loss, estimated blood loss, and Harris score) were expressed as mean differences (MD) with their respective 95% CIs. Discontinuous outcomes (the rates of need for transfusion) were expressed as risk difference (RD) with 95% CIs. Statistical significance was set at *P* < 0.05. To summarize findings across the trials, Software RevMan 5.3 (The Cochrane Collaboration, Oxford, UK) was used for meta-analysis. Statistical heterogeneity was tested using the Chi-squared test and *I*^2^ statistic. Chi-squared test results with *P* > 0.1 were considered suggestive of statistical heterogeneity. Heterogeneity was also assessed by the *I*^2^ statistic, which describes the percentage of total variation across studies that is caused by heterogeneity rather than chance. An *I*^2^ statistic value of N 50% was considered to indicate substantial heterogeneity. Depending on the heterogeneity, a meta-analysis was performed using fixed effect or random effect models. When there was no statistical evidence of heterogeneity, a fixed effect model was adopted; otherwise, a random effect model was chosen.

## RESULTS

### Search Result

**FIGURE 1:**
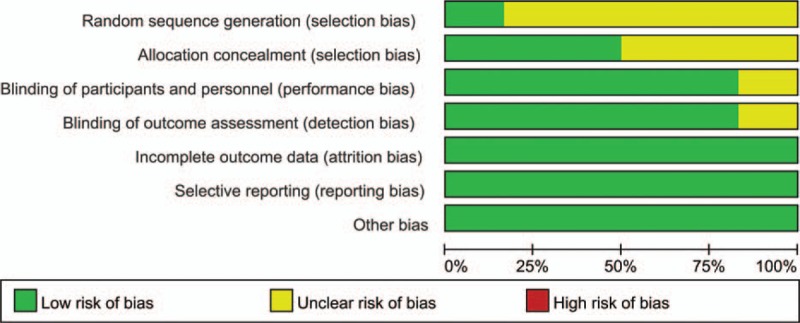
Risk of bias. Each risk-of-bias item presented as percentages across all included studies, which indicated the proportion of different levels of risk of bias for each item.

**FIGURE 2:**
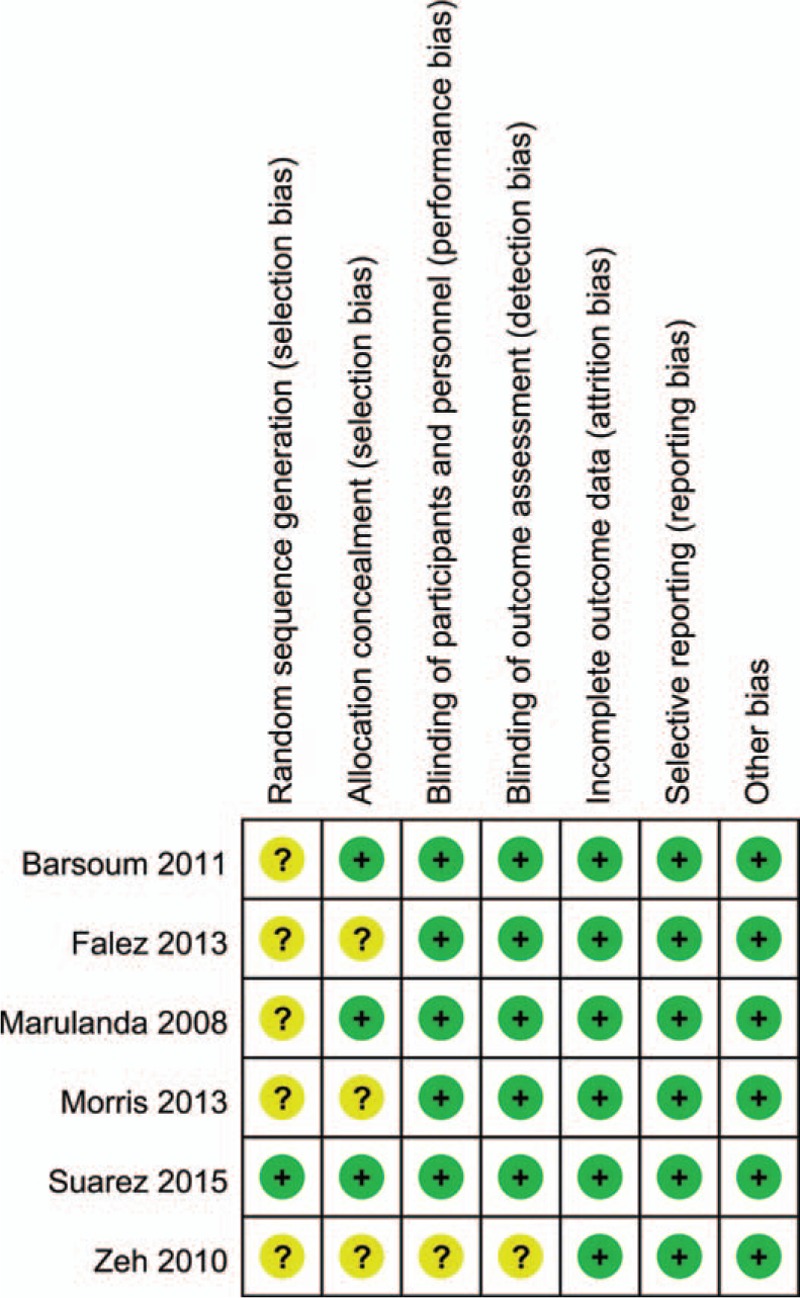
Methodological quality of the included studies. This risk-of-bias tool incorporates assessment of randomization (sequence generation and allocation concealment), blinding (participants, personnel, and outcome assessors), completeness of outcome data, selection of outcomes reported, and other sources of bias. The items were scored with “yes,” “no,” or “unsure.”

In the initial search, we identified 422 potentially relevant studies, of which 80 duplicates were removed by Endnote Software. According to the inclusion criteria, 336 studies were excluded after reading the titles and abstracts. Finally we included 6 clinical trials with 677 patients (677 hips) in the meta-analysis (Figure 1).^9–14^ The characteristics of the included studies are shown in Table 1. Of the included studies, a total of 677 THAs were performed and the number of bipolar sealer and standard electrocautery was 334 and 343, respectively. All included articles were in English and were published between the years 2008 and 2015. All participants in the 6 studies were elderly patients aged between 55.4 and 68.3 years who were planning to undergo THA. Risk of bias can be seen in Figures 1 and 2.

**TABLE 1:**
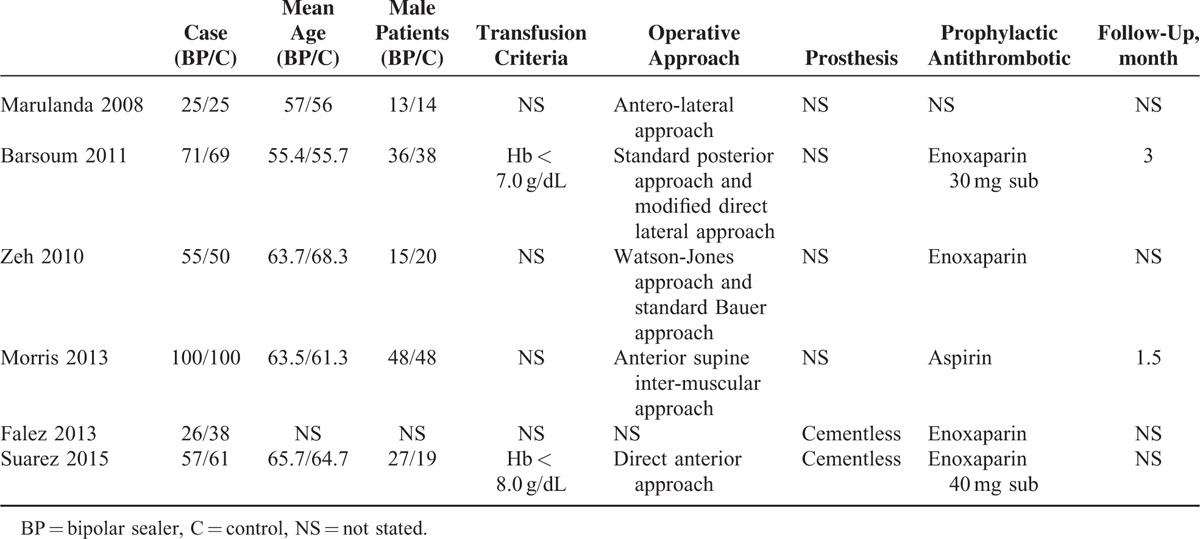
The General Characteristic of the Included Literatures

### Results of Meta-Analysis

#### Need for Transfusion

**FIGURE 3:**
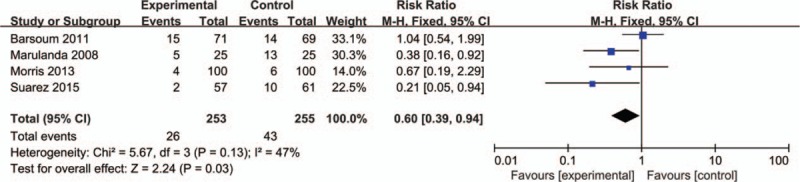
Forest plot comparing the need for transfusion in bipolar sealer group and control group Hb Drop.

Only 4 studies with 508 THAs reported the need for transfusion postoperatively. The meta-analysis revealed that bipolar sealer can decrease the need for transfusion for patients who underwent THA (relative risk [RR] = 0.60; 95% CI 0.39–0.94; *P* = 0.03, Figure 3).

### Hb Drop

**FIGURE 4:**
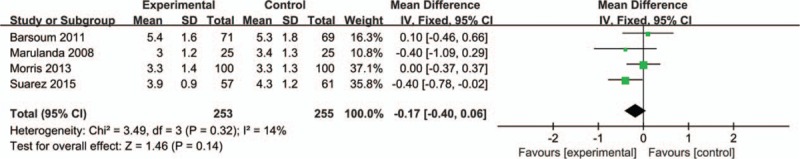
Forest plot comparing Hb drop in bipolar sealer group and control group.

A total of 4 studies (508 patients) reported the Hb drop of the 2 groups. There was no significant difference between the bipolar sealer and control group (MD = −0.17; 95% CI −0.40–0.06; *P* = 0.14, Figure 4).

#### Blood Loss in Drainage, Hidden Blood loss, Estimated Blood Loss, Total Blood Loss, and Intraoperative Blood Loss

**FIGURE 5:**
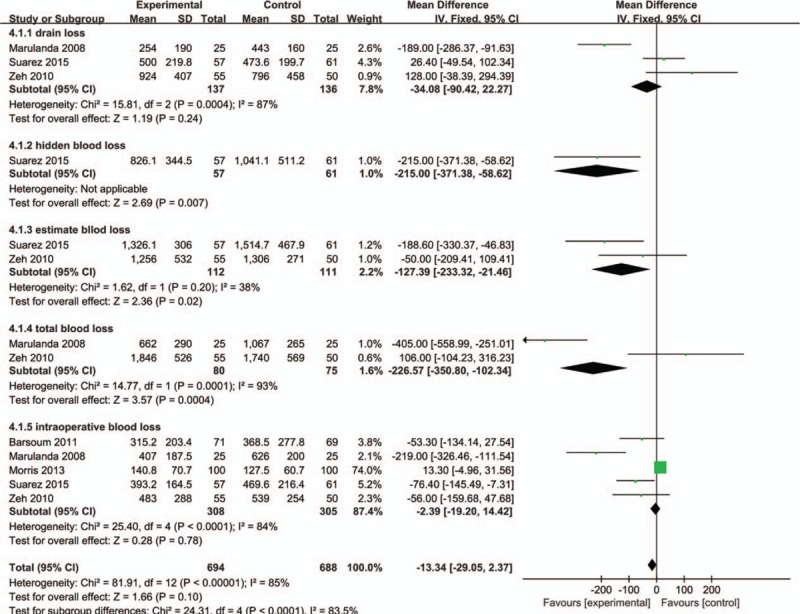
Forest plot comparing the total blood loss, blood loss in drainage, hidden blood loss, and intraoperative blood loss in bipolar sealer group and control group.

**FIGURE 6:**
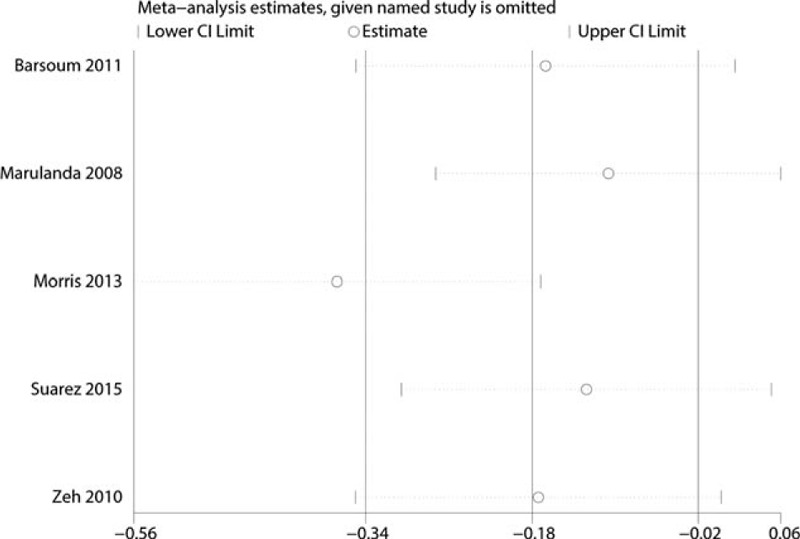
The sensitivity analysis of the included studies for intraoperative blood loss.

There was no significant difference between the groups (Figure 5) (MD = −34.08; 95% CI −90.42–22.27; *P* = 0.24). The result of this meta-analysis revealed that bipolar sealer can decrease the intraoperative blood loss (Figure 5) (MD = −2.39; 95% CI −19.20–14.42; *P* = 0.78). Since the heterogeneity between the studies is large (χ^2^ = 25.40, *P* < 0.00001, *I*^2^ = 84%) and thus sensitivity analysis was conducted to seek the source of the heterogeneity. The result of sensitivity analysis indicated that Morris study influence the final result (Figure 6).

The meta-analysis revealed bipolar sealer can reduce the hidden blood loss (Figure 5) (MD = −215.00; 95% CI −317.38 to −58.62; *P* = 0.007), total blood loss (MD = −226.57; 95% CI −50.80–102.34; *P* = 0.0004), and estimated blood loss (MD = −127.39; 95% CI −233.32 to −21.46; *P* = 0.02).

### Length of Hospital Stay

**FIGURE 7:**
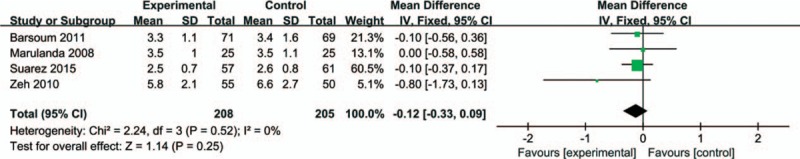
Forest plot comparing the length of hospital stay in bipolar sealer group and control group. Operating time.

A total of 4 studies (413 patients) provided data on the length of hospital stay. The meta-analysis revealed that there was no significant difference between the 2 groups regarding the length of hospital stay (Figure 7) (MD = −0.12; 95% CI −0.33–0.09; *P* = 0.25).

### Operating Time

**FIGURE 8:**
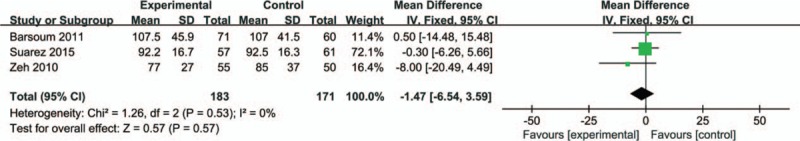
Forest plot comparing the operating time between bipolar sealer group and control group. Rate of infection.

A total of 3 studies (354 patients) provided data on the operating time. The result of meta-analysis revealed that there was no significant difference between the 2 groups regarding the operating time (Figure 8) (MD = −1.47; 95% CI −6.54–3.59; *P* = 0.57).

### Rate of Infection

**FIGURE 9:**
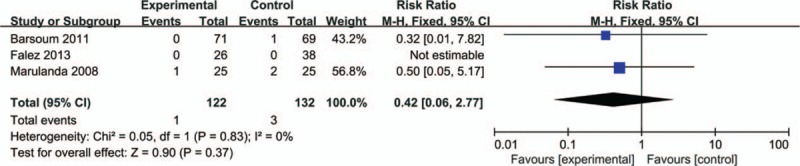
Forest plot comparing the rate of infection between bipolar sealer group and control group. Harris score.

A total of 3 studies (254 patients) provided data of rate of infection. The result of meta-analysis revealed that there was no significant difference between the 2 groups regarding the rate of infection (Figure 9) (RR = 0.42; 95% CI 0.06–2.77; *P* = 0.37).

### Harris Score

**FIGURE 10:**
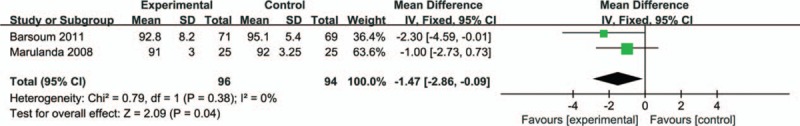
Forest plot comparing the Harris score between bipolar sealer group and control group.

Two studies (190 patients) provided data on Harris score. The result of meta-analysis revealed that there was no significant difference between the 2 groups regarding the Harris score (MD = 0.42; 95% CI 0.06–2.77; *P* = 0.37, Figure 10).

## DISCUSSION

The blood loss during THA is large and difficult to manage, since the blood products is limited and large cost, so an economic and safety blood strategy can fulfill the clinical demand. Currently, tranexamic acid, fibrin sealant, thrombin-based hemostatic agent, and bipolar sealer are used to reduce the postoperative blood loss.^15–20^ In addition, intraoperative blood management is another important aspect to decrease the patients need for transfusion. Bipolar sealer is mainly administrated to reduce the operative blood loss; however, the effect of hemostasis is controversial. So, a meta-analysis to further identify the effect and safety of bipolar sealer of hemostasis during THA is necessary.

The current study is mainly to compare the efficacy and safety of bipolar sealer in management of blood loss after THA. Further, the most significant finding of this meta-analysis is that bipolar sealer can decrease the need for transfusion, total blood loss, and hidden blood loss. Notably, this meta-analysis did not show any significant differences in the effects of Hb decrease, rate of infection, operating time, and Harris score. Additionally, there were no significant differences between groups in terms of the blood loss in drainage and estimated blood loss. Finally, the length of hospital stay between the 2 groups was not significantly different. The clinical effect of this meta-analysis is that administration bipolar sealer during operation can decrease the intraoperative blood loss.

The bone was a special tissue that contained most of inorganic components that is very poor conductor to electricity. That is another reason that leads to the large amount intraoperative blood loss since the electrocautery is difficult to manage the blood loss. Rosencher et al^3^ conducted a meta-analysis and found that the blood loss in primary THA can be reached to as large as 1944 mL. Bipolar sealer has been used for THA, total knee arthroplasty, and spine surgery to reduce the blood loss during surgery for many years; however, there is no consensus about the efficacy and safety of the hemostasis in THA. Although a previous meta-analysis has been conducted to compare the efficacy of bipolar sealer in reducing blood loss after THA.^21^ In his study, the hidden blood loss and estimated blood loss were not to be compared. The special of our study is that more RCT included and the outcome of hidden blood loss was compiled in our meta-analysis. And the shortcoming of the previous study is they do not distinguish the cemented and noncemented prosthesis. From the result of this meta-analysis, bipolar sealer can reduce the number of patients that need for transfusion and total blood loss. This result can be contradicted from the previous study. The large heterogeneity between the studies will decrease the reliability of the final conclusion. The different operative approach will have influence on the final hip function. In the included studies, one study performed THA in antero-lateral approach,^11^ one study administrated standard posterior approach and modified direct lateral approach,^9^ one study in Watson-Jones approach and standard Bauer approach,^14^ one study performed in Anterior supine inter-muscular approach,^12^ one study not related to the approach,^10^ and one study performed in direct anterior approach.^13^

As for the need for transfusion, the result of our meta-analysis showed that bipolar sealer can reduce the need for transfusion and the difference was statistically significant (RR = 0.60; 95% CI 0.39–0.94; *P* = 0.03); however, this result should be treated carefully since the *P* value is 0.03. This result also means the bipolar sealer may reduce the economic cost that applied into the transfusion. Kamath et al^22^ applied the bipolar sealer in simultaneous bilateral THA, the conclusion drawn from the article is that the bipolar sealer with the goal of less blood loss must come with the additional expense associated with its use. Meanwhile Derman and Lee^23^ use of a saline-coupled bipolar sealing device in patients with infected total knee arthroplasties is not clinically or economically justified. Wang et al^24^ reported that bipolar sealer can reduce the blood loss and the need for transfusion for 0.7 U red blood cell in posterior spinal fusion for degenerative lumbar scoliosis.

As regarded to the Hb drop, there is no significant difference between the bipolar sealer group and control group (MD = −0.17; 95% CI −0.40−0.06; *P* = 0.14). Meanwhile, bipolar sealer can reduce total blood loss and hidden blood loss during THA. Falez et al^10^ compared the bipolar sealer versus fibrin sealant and find bipolar sealer remarkable reduce the blood loss at 48 hours and fibrin sealant reduce at every time interval. However, all the included studies do not reported the incurrence of severe burns, severe tissue necrosis, and operating room fires. Hidden blood loss during THA takes a great proportion of total blood loss and the mechanism of hidden blood loss is not clear. Only one study reported the hidden blood loss during THA, bipolar sealer can reduce hidden blood loss and total blood loss. Despite this hemostasis effect of bipolar sealer, tranexamic acid has been applied into clinician used for many years and the lower cost appeals the clinician. A study has reported that the therapeutic dose of TA set at 10 mg/kg at induction of anesthesia will cost about 8€; and an Aquamantys Bipolar Sealer set costs up to 1440,00€.^25^ All of the included studies do not compare the cost of bipolar sealer and standard electrocoagulation, so the balance between the decrease of transfusion and the additional cost is unknown to us.

Ackerman et al^26^ use medical resource hospital administrative database to analyze the cost of bipolar sealer and draw a conclusion that bipolar sealer can decrease the need for transfusion without increasing total hospital cost. There have been referenced that the use of this bipolar sealer allows for a decrease in postoperative pain and swelling, thus lead to the quickly recovery from total knee arthroplasty.^27^ In our study, there is no significant difference between the Harris score after 6 months, this means that bipolar sealer is not helpful for long-term recovery after THA. The results can be explained for the not long enough follow-up. So, a long-term follow-up visit is still needed to identify the effect of bipolar sealer.

This meta-analysis has several potential limitations.

1. Only 6 RCTs compared bipolar sealer with standard electric coagulation for the blood loss management after THA and finally included in our meta-analysis, meaning that the limited sample analyzed was insufficient.
2. Four studies did not report the random sequence generation and this will have selection bias to the final result.
3. The sample in each study is not large enough and this will influence the final result.
4. There may be publication bias as in all meta-analyses, which could also have affected the results.
5. The follow-up in some studies is not clear, and this will under-reporting the occurrence of complications.

## CONCLUSION

The present meta-analysis indicated that bipolar sealer can significantly reducing the need for transfusion, estimated blood loss, and total blood loss, there is no significant difference between the Hb drop, intraoperative blood loss, and blood loss in drainage. What is more, there is no benefit for the bipolar sealer for the recovery of hip function and the risk for infection. To be use the bipolar sealer or not, it is still decided by the economic cost and the rate for transfusion, and this will need more clinical trials to draw a definite conclusion.
